# The effects of serum cholesterol, LDL, and HDL levels on gallstone cholesterol concentration

**DOI:** 10.12669/pjms.291.2798

**Published:** 2013

**Authors:** S. Selcuk Atamanalp, M. Sait Keles, R. Selim Atamanalp, Hamit Acemoglu, Esra Laloglu

**Affiliations:** 1 S. Selcuk Atamanalp, Professor, Department of General Surgery, Faculty of Medicine, Ataturk University, Erzurum, Turkey.; 2 M. Sait Keles, Professor, Department of Biochemistry, Faculty of Medicine, Ataturk University, Erzurum, Turkey.; 3 R. Selim Atamanalp, English Medicine Section, Class 1, Faculty of Medicine, Ataturk University, Erzurum, Turkey.; 4 Hamit Acemoglu,Associate Professor, Department of Medical Education, Faculty of Medicine, Ataturk University, Erzurum, Turkey.; 5 Dr. Esra Laloglu, Department of Biochemistry, Faculty of Medicine, Ataturk University, Erzurum, Turkey.

**Keywords:** Cholesterol, LDL, HDL, Gallstone

## Abstract

***Objective:*** Gallbladder stones are common in the Western world, and 70% to 80% of gallstones are cholesterol stones. This study investigates the effects of serum cholesterol, LDL, and HDL levels on gallstone cholesterol concentration.

***Methodology:*** The gallstones of 75 patients with cholelithiasis were examined using spectrophotometry.

***Results:*** High serum cholesterol and LDL levels were associated with high cholesterol stone rates (86.7% vs. 40.0%, P < 0.001; 75.0% vs. 48.9%, P < 0.05, respectively). Similarly, high serum cholesterol and LDL levels were correlated with high gallbladder stone cholesterol concentrations (63.6% vs. 44.4%, P < 0.001; 62.3% vs. 46.0%, P < 0.001, respectively). In contrast, low serum HDL levels do not seem to affect the occurrence of gallbladder cholesterol stones (60.0% vs. 58.3%, respectively, P > 0.05) or gallbladder stone cholesterol concentrations (50.8% vs. 52.4%, respectively, P > 0.05).

***Conclusion:*** The relationship between cholesterol, LDL, and HDL levels and cholesterol gallstone formation is multifactorial and complex and is also dependent on other individual properties.

## Introduction

 Gallbladder stones are common in the Western world with an incidence of 1.4 per 100 person-years.^1^ Gallstones are classified by their cholesterol content as either cholesterol or pigment stones, and most stones are made of mixed contents, while pure stones are rare.^2^ The stones with cholesterol concentrations higher than 50% are considered to be cholesterol stones,^3^ and 70% to 80% of gallstones are cholesterol stones.^4^^,^^5^

 Cholesterol is a lipid. Its major synthesis site is the liver, and its only excretion site is the biliary system.^2^ The pathogenesis of cholesterol gallstones is multifactorial and complex. The known factors associated with cholesterol gallstones include cholesterol hypersecretion and supersaturation, bile salt and phospholipid concentrations, crystal nucleation, gallbladder dysmotility and gallbladder absorption and secretion functions.^2^^,^^3^^,^^6^^,^^7^ Low-density lipoproteins (LDLs) and high-density lipoproteins (HDLs) are plasma lipids, and their main function is to transport cholesterol. LDL transports cholesterol from the liver to the peripheral tissues, and HDL transports cholesterol from the peripheral tissues to the liver.^2^

 This study investigated the effects of serum cholesterol, LDL, and HDL levels on the cholesterol concentration of gallstones in patients with cholelithiasis.

## Methodology

 This study was conducted over a 12-month period from March 2011 to March 2012. A total of 75 patients with cholelithiasis from whom gallstones were obtained after cholecystectomy were included in this study. The study was approved by the Institutional Ethics Committee. The clinical and laboratory records of the patients were examined for the age, gender, and serum cholesterol, LDL, and HDL levels.

 At least one gallstone was obtained from each patient, and the gallstones were stored in normal room conditions. After chemical blanking, 282 μL of a cholesterol measurement reagent (Cobas Cholesterol Gen 2)^8^ and 12 μL of a cholesterol calibrator solution (containing 165 mg/dL cholesterol) were transferred into an ultraviolet spectrophotometric tube and measured in a spectrophotometer (Beckman DU 530) at a wavelength of 505 nm. A 30 mg sample of the powdered stone was placed into a test tube, 3 mL of chloroform solution was added, and the tube was boiled for two minutes to resolve the cholesterol. Similarly, 282 μL of a cholesterol measurement reagent and 12 μL of boiled solution (instead of the cholesterol calibrator solution) were added to an ultraviolet spectrophotometric tube, and the spectrophotometric measurement was repeated.

 The cholesterol concentration of the stone was determined by comparing the measured values of the cholesterol calibrator solution and the boiled solution. To convert the measured mg/dL value to mg/mL, it was divided by 100 because the chloroform solution was measured in mL. The calculated value was then multiplied by 3 because 3 mL of chloroform solution was used. The value was divided by 30 because the cholesterol concentration was calculated using a 30 mg sample of the biliary stone. Thus, the cholesterol concentration of the stone was determined in mg of cholesterol per mg of stone; the cholesterol percentage in the stone was also calculated.^2^^,^^9^ The stones with cholesterol concentrations higher than 50% were considered to be cholesterol stones.^3^

The chi-square test and Student’s t test were used for the statistical analyses, and statistical significance was set at P<0.05.

**Table-I:** The cholesterol stone numbers and stone cholesterol concentrations according to the serum cholesterol, LDL and HDL levels.

*Group*	*Level*	*Total*	*Cholesterol stone number*	*Chi-square test*	*Stone cholesterol concentration*	*Student’s t test*
Cholesterol	High	30	26 (86.7%)	P< 0.001	63.6±16.5%	P< 0.001
	Normal or low	45	18 (40.0%)		44.4±16.0%	
LDL	High	28	21 (75.0%)	P< 0.05	62.3±17.0%	P< 0.001
	Normal	47	23 (48.9%)		46.0±17.0%	
HDL	Low	15	9 (60.0%)	P> 0.05	50.8±20.7%	P> 0.05
	Normal or high	60	35 (58.3%)		52.4±18.3%	

## Results

 The mean age of the 75 included patients was 50.4 years (range: 16-81 years), and 58 patients (77.3%) were female. Cholesterol stones were identified in 44 patients (58.7%), and the stone cholesterol concentrations were between 9% and 91% (mean: 52.1%).

 The number of cholesterol stones and the stone cholesterol concentrations according to the serum cholesterol, LDL and HDL levels are given in Table-I. The serum cholesterol levels were between 58 and 274 mg/dL (mean: 186.0 mg/dL). Thirty patients (40.0%) had a high serum cholesterol level (normal range: 120-200 mg/dL), and they had a significantly higher cholesterol stone rate when compared with the patients with a normal or low serum cholesterol level (86.7% vs. 40.0%, respectively, P<0.001). Similarly, the mean stone cholesterol concentration of the patients with high serum cholesterol was significantly higher than that of the others (63.6% vs. 44.4%, respectively, P<0.001).

 The serum LDL levels were between 12 and 215 mg/dL (mean: 121.8 mg/dL). Twenty-six patients (37.3%) had a high serum LDL level (normal range: 0-130 mg/dL), and they had a higher cholesterol stone rate when compared with the patients with a normal serum LDL level (75.0% vs. 48.9%, respectively, P<0.05). Similarly, the mean stone cholesterol concentration of the patients with a high serum LDL level was significantly higher than the others (62.3% vs. 46.0%, respectively, P<0.001).

 The mean HDL levels were between 16 and 113 mg/dL (mean: 52.8 mg/dL). Fifteen patients (20.0%) had a low serum HDL level (normal range: 40-60 mg/dL). There was no statistically significant difference in the cholesterol stone rate between patients with a low serum HDL level and patients with a normal or high serum HDL level (60.0% vs. 58.3%, respectively, P<0.05). Similarly, there was no statistically significant difference in the mean stone cholesterol concentrations in patients with a low serum HDL level and the other patients (50.8% vs. 52.4%, respectively, P>0.05).

## Discussion

 Cholesterol is the most prevalent substance in gallstones, and it is detected in 95.0% of samples.^10^ The data from the present study support these findings. Cholesterol stones are the most common type of gallstones, and they have been identified as 67.5% of gallstones by Mazlum et al.^11^, 70% by Van Erpecum,^3^ and 75% by Whiting et al.^5^ Similarly, we found that 58.7% of the sample gallstones were cholesterol stones. Otherwise, the mean gallstone cholesterol concentration was reported to be 25% by Angwafo et al.^12^ 59.7% by Chandran et al.^9^ and 60.8% by Jaraari et al.^13^ We determined that the mean stone cholesterol concentration was 52.1%.

 Khairy et al.^14^ reported a positive association between high serum cholesterol levels and cholesterol gallstone development. Similarly, Halldestam et al.^1^ and Andreotti et al.^15^ argued that high serum cholesterol levels are positively correlated with gallstone disease. Our study demonstrated similar results, and positive correlations between a high serum cholesterol level and a high cholesterol stone rate and a high stone cholesterol concentration were shown. In contrast, Thijs et al.^16^ found an inverse correlation between the serum cholesterol level and gallstone risk.

 A high serum LDL level was described as a marker for increased risk of cholesterol gallstone disease by Fu et al.^17^ Similarly, Han et al.^18^ and Fu et al.^19^ revealed a positive correlation between a high serum LDL level and cholesterol gallstone development, and Halldestam et al.^1^ demonstrated a relationship between a high serum LDL level and gallstone disease. The results of our study support these results; we observed positive correlations between a high serum LDL level and a high cholesterol stone rate and a high stone cholesterol concentration. In contrast, Andreotti et al.^15^ and Tang^20^ reported an inverse correlation between the serum LDL level and gallstone risk.

 Andreotti et al.^15^ reported a 4.2-fold increased risk of gallstone disease in individuals with a low serum HDL level. Similarly, Fu et al.^17^ identified a positive correlation between a low serum HDL level and cholesterol gallstone development, while Thijs et al.^16^ and Tang^20^ revealed a relationship between a low serum HDL level and gallstone disease. By contrast, Halldestam et al.^1^ and Fu et al.^19^ did not observe a positive correlation between a low serum HDL level and gallstone development. The results of our study demonstrated similar results to these reports, and no association was observed between the serum HDL level and the cholesterol stone rate or the stone cholesterol concentration.

 High serum cholesterol, high serum LDL, and low serum HDL levels may be expected to increase cholesterol excretion with bile and cause cholesterol gallstone disease. However, although our results demonstrated the expected relationships between serum cholesterol and LDL levels and cholesterol gallstone formation, serum HDL levels were not associated with cholesterol gallstone formation. The relationship between serum cholesterol, LDL, and HDL levels and cholesterol gallstone formation is multifactorial and complex and is also dependent on other individual properties.
